# The Role of Cyclooxygenase-2 in Cell Proliferation and Cell Death in Human Malignancies

**DOI:** 10.1155/2010/215158

**Published:** 2010-03-17

**Authors:** Cyril Sobolewski, Claudia Cerella, Mario Dicato, Lina Ghibelli, Marc Diederich

**Affiliations:** ^1^Laboratoire de Biologie Moléculaire et Cellulaire du Cancer, Hôpital Kirchberg, 9 rue Edward Steichen, 2540 Luxembourg, Luxembourg; ^2^Dipartimento di Biologia, Università di Roma di Roma Tor Vergata, Via Ricerca Scientifica snc, 00133 Rome, Italy

## Abstract

It is well admitted that the link between chronic inflammation and cancer involves cytokines and mediators of inflammatory pathways, which act during the different steps of tumorigenesis. The cyclooxygenases (COXs) are a family of enzymes, which catalyze the rate-limiting step of prostaglandin biosynthesis. This family contains three members: ubiquitously expressed COX-1, which is involved in homeostasis; the inducible COX-2 isoform, which is upregulated during both inflammation and cancer; and COX-3, expressed in brain and spinal cord, whose functions remain to be elucidated. COX-2 was described to modulate cell proliferation and apoptosis mainly in solid tumors, that is, colorectal, breast, and prostate cancers, and, more recently, in hematological malignancies. These findings prompt us to analyze here the effects of a combination of COX-2 inhibitors together with different clinically used therapeutic strategies in order to further improve the efficiency of future anticancer treatments. COX-2 modulation is a promising field investigated by many research groups.

## 1. Introduction: Inflammation and Cancer are Linked

Inflammation is the major reaction of natural immunity with the goal to defend the organism against pathogens. It can be induced upon bacterial infections by compounds including lipopolysaccharides, as well as by viruses, which are detected by Toll-like receptors (TLRs), expressed by immune cells like macrophages. Besides, inflammation can be triggered by physical injuries (i.e., UV) or chemical compounds (i.e., reactive oxygen species) [1]. The activation of specific receptors triggers intracellular signals (i.e., NF*κ*B, p38, or MAPKsmediated), which regulate pro-inflammatory cytokine expression, such as interleukin 1 beta (IL1*β*), tumor necrosis factor alpha (TNF*α*), interleukin 6 (Il6), together with chemokines and cell adhesion proteins [1], in turn, leading to the recruitment and the activation of immune cells. 

Several diseases are associated to chronic inflammation, such as osteoarthritis, Crohn's disease, and cancer [2]. Although the first evidence of a connection between inflammation and cancer dates back to more than a century ago [3], only recently, this link has been further investigated, thus evidencing that the incidence of several cancers is tightly associated to inflammation such as colon, breast, and prostate cancers [4–6]. This hypothesis is supported by the findings that the tumor microenvironment is characterized by the infiltration with different types of immune cells (*i.e*., dendritic cells, lymphocytes, and macrophages) responsible for the release of cytokines [1]. The role of these cytokines in tumor incidence has been established in many studies. For example, the overexpression of TNF*α* in transgenic mice bearing a lung tumor is associated with an increase of the size of the tumor [7]. Moreover, a chronic intake of nonsteroidal antiinflammatory drugs (NSAIDs) leads to a significant reduction in the incidence of such tumors. Colorectal cancer (CRC), which remains an important cause of death in the industrialized world, is one of the most characterized types of tumor that benefits from treatment by NSAIDs [8]. Interestingly, chronic use of aspirin is reported to reduce the relative risk of CRC by about 50% [9]. Familial adenomatous polyposis, an inherited form of colon cancer, is characterized by the development of preneoplastic polyps. At the molecular level, this disease is caused with a mutation of a tumor suppressor gene called Adenomatous polyposis coli (APC). It has been shown that the use of NSAIDs, like sulindac, as a chemopreventive treatment, is able to decrease the incidence of polyp formation [10]. Similar results were obtained with celecoxib [11], which is now approved by the Food and Drug Administration's Oncologic Drugs Advisory Committee as an adjuvant in FAP therapy.

A body of evidence indicates a role for inflammation in the development/modulation of different steps of cancer progression. Inflammation may play a role in tumor initiation by triggering the production of reactive oxygen species (ROS), responsible for DNA damage, thus increasing the rate of mutations [12]. It may also be implicated in tumor promotion, where inflammation triggers the secretion of growth factors, such as the epithelial (EGF) and fibroblast growth factors (FGF). These, in turn, favor the proliferation of initiated tumor cells by determining an imbalance between cell proliferation and cell death stimuli [6], due to the activation of different cell survival pathways [7]. 

Besides, the different cytokines produced during inflammation (*i.e*., TNF*α*, IL1*β*, IL6, and IL8) can also activate several survival pathways, thus leading to an escape of tumor cells from cell death. Well known is the case of TNF*α*, produced by tumor and immune cells, which leads to the survival of cancer cells by the upregulation of antiapoptotic proteins, that is, Bcl-2 [13–15],* via* the activation of the nuclear factor kappa B (NF*κ*B) [16]. The modulation of pro-survival pathways or anti-apoptotic proteins makes the expression/activation of such proinflammatory mediators also a determining factor in chemoresistance. A constitutive activation of such proinflammatory factors has been frequently found in many cancers, such as hepatocellular carcinoma [17], prostate cancer [18], as well as chronic and acute myeloid leukemia [19], where it is frequently associated with a bad prognosis. In these instances, the modulation of Bcl-2 anti-apoptotic family members has been frequently shown [13–15, 20].

Amongst the different mediators of inflammation, the cyclooxygenases (COXs) clearly appear to be implicated in cancer. This review focuses on COX-2, the inducible form, normally induced and implicated in inflammation, and intends to analyze what is currently known about the link between COX-2 and cancer, in terms of effects on cell proliferation and cell death. In this view, we will focus our attention on studies analyzing the effects of COX-2 inhibitors on cancer cells, when used alone as well as in combination with therapeutic approaches, including radiotherapy, chemotherapeutic agents, and photodynamic therapy. Finally, we will consider the relevance of COX-2-independent effects.

## 2. The Cyclooxygenase Enzyme Family

**Figure 1:**
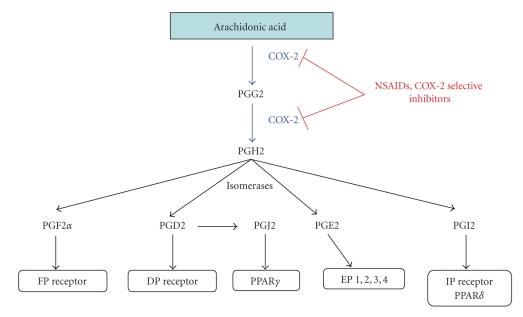
*Metabolism of arachidonic acid by COX-2 and receptors implicated in response to prostaglandins* (according to Chandrasekharan et al. [21]). Prostaglandins act through different receptors to mediate their effects. PGE2 is able to bind four receptors (EP1, 2, 3, and 4). These receptors do not possess the same ligand affinity and their expression is tissuedependent. The different receptors are associated with different intracellular pathways. Most of these receptors are localized in the plasma membrane but nuclear receptors PPAR*γ* can also bind PGJ2. Abbreviation: COX-2, cyclooxygenase-2; PG, prostaglandin; FP, prostaglandin F receptor; DP, prostaglandin D receptor; EP, prostaglandin E receptor; IP, prostaglandin I receptor; PPAR, peroxisome proliferator-activated receptor; NSAIDs, nonsteroidal anti-inflammatory drugs.

Cyclooxygenases (or prostaglandin H synthases), commonly referred to as COXs, are a family of myeloperoxidases located at the luminal side of the endoplasmic reticulum and nuclear membrane [21], which catalyze the rate-limiting step of prostaglandin biosynthesis from arachidonic acid [21]. These enzymes act by two coupled reactions. The first one is the conversion of arachidonic acid released from the plasma membrane by phospholipase A2 to prostaglandin G2 by the cyclooxygenase activity. The second reaction is mediated by the peroxidase activity and leads to the conversion of prostaglandin G2 to prostaglandin H2. Then, different synthases convert prostaglandin H2 to prostaglandin D2, F2*α*, E2, I2, and thromboxane A2 (Figure 1).

Prostanoids (prostaglandins and thromboxanes) are immediately released from the cells, where it is believed that they act locally in an autocrine and paracrine manner through different receptors activating different intracellular pathways still to be completely elucidated (Figure 1) [22]. Prostaglandins, specifically, are important for physiological functions like vasodilatation (PGD2, PGE2, PGI2), gastric cytoprotection (PGI2), maintenance of renal homeostasis, and platelet aggregation. Besides, prostaglandins play a major role in mediating fever (PGE2), pain sensitivity, and inflammation [21].

So far, three isoforms of COXs have been identified. Cyclooxygenase-1 (COX-1) is a glycoprotein of 71kDa, which is constitutively expressed in different tissues. COX-1 is encoded by a gene on chromosome 9 and plays a role in tissue homeostasis by modulating several cellular processes ranging from cell proliferation to angiogenesis or platelet aggregation due to thromboxane production [21].

Cyclooxygenase-2 (COX-2) is the inducible isoform, which is regulated by growth factors and different cytokines such as IL1*β*, IL6, or TNF*α* [23], therefore overexpressed during inflammation. The COX-2 gene is located on chromosome 1 and its promoter displays an NF*κ*B response element as well as other cytokine-dependent (i.e., IL6) response elements [21]. The protein shows a 60% homology with COX-1 [24]; in addition, COX-2 presents a C-terminal extension and a different binding site for NSAIDs, which makes COX-2 a preferential target compared to COX-1, thus being specifically inhibited at lower doses [25].

Finally, COX-3 has been identified as a splice variant of COX-1, and it is present mainly in brain and spinal cord [26, 27]. Currently, the role of COX-3 is not known. Some pieces of evidence suggest a possible role in pain sensitivity, based on studies focused on the mechanism of action of acetaminophen (paracetamol), recently evoked as a selective inhibitor of COX-3 [28]. However, this hypothesis is debated because other findings argue that acetaminophen targets at the same time COX-2 [29].

## 3. COX-2 As a Tumor Promoter and a Good Candidate for Cancer Therapy

Overexpression of COX-2 has been detected in a number of tumors, such as colorectal breast as well as pancreatic and lung cancers [2, 30–32], where it correlates with a poor prognosis. Moreover, overexpression of COX-2 has been reported in hematological cancer models such as RAJI (Burkitt's lymphoma) and U937 (acute promonocytic leukemia) [33, 34] as well as in patient's blast cells [32, 34]. Data suggested that COX-2 may play a role in different steps of cancer progression, by increasing proliferation of mutated cells [30], thus favoring tumor promotion as well as by affecting programmed cell death and affecting the efficacy of anticancer therapies [35–39] to be, finally, implicated in metastasis formation, for example, by affecting apoptosis induced by loss of cell anchorage (anoikis) [40].

COX-2 induction or overexpression is associated with an increased production of PGE2, one of the major products of COX-2 which is known to modulate cell proliferation, cell death, and tumor invasion in many types of cancer including colon, breast, and lung. Prostaglandin E2 acts through different membrane receptors called EP receptors (EP1, EP2, EP3, and EP4) [41]. These receptors are all located on the cell surface and characterized by seven-transmembrane domains, and rhodopsin-type G protein-coupled receptors, but trigger different signaling pathways. Thus, it is known that EP1 signaling acts through phospholipase C/inositol triphosphate signaling, leading to intracellular mobilization of calcium. EP2 and EP4 receptors are coupled with G proteins which activate adenylate cyclase, leading to an increase of intracellular cAMP [41]. cAMP is then able to activate kinases such as protein kinase A (PKA) or PI3K for example, and also GSK3 leading to an activation of *β*-catenin, a pathway regulating cell proliferation [42, 43]. In contrary to EP2 and EP4, EP3 is coupled with Gi protein, leading to an inhibition of adenylate cyclase, and thus a decrease of cAMP inside the cells [41]. The differential expression of these different receptors according to the cell type may explain the diverse and antagonist effects of PGE2 described in literature.

Until now, there are multiple evidences about the role of PGE2 in tumorigenesis in some cancers. These evidences are mostly described for adherent tumors while this link is poorly understood for hematopoietic malignancies such as leukemia or lymphoma. Indeed, several papers have reported that PGE2 is the most important prostaglandin produced during colorectal carcinogenesis [44]. Moreover, it is known that the level of PGE2 increases in a size-dependent manner in Familial Adenomatous Polyposis (FAP) patients [45], suggesting a correlation between tumor growth and prostaglandin biosynthesis. Tumorigenesis is characterized by a disequilibrium between cell proliferation and cell death. PGE2 is able to inhibit apoptosis in human colon cancer cells. It has been demonstrated that PGE2 can upregulate the level of the anti-apoptotic protein Bcl-2 in HCA-7 cells (adenocarcinoma), which produce significant amounts of PGE2. This paper described a modulation of the MAPK pathway that precedes the upregulation of Bcl-2 [46]. PGE2 can mediate its effect through EGF receptor, leading to MAPK activation. The ability of PGE2 to modulate tumor progression in colorectal cell has been shown in other models of colon cancer such as HT-29 cells that express EP receptors. In this cell type, PGE2 is associated with an increase of cAMP through EP4 receptor. The effect can be reversed by L-161982, an antagonist of EP4 [47]. Moreover, PGE2 transactivates EGFR by triggering the release of amphiregulin, a well-known EGFR ligand [48]. SC-236, an inhibitor of COX-2, is able to inhibit cell proliferation of HT-29 cells and this effect is greater in combination with an amphiregulin neutralizing antibody [47]. In this cell line, the expression of amphiregulin is correlated to the expression of COX-2.

The transactivation of EGFR by PGE2 can lead also to AKT activation, which is a well-known survival pathway [49]. This effect was well described in a study by Tessner et al. [50] demonstrating that 16,16-dimethyl PGE2 (dmPGE2) inhibits radiation-induced apoptosis in the mouse intestinal epithelium. Using HCT-116 cell line as a model to reflect the effect on mouse small intestine, it has been shown that the anti-apoptotic effect of dmPGE2, which is known to bind EP2, was tightly related to AKT phosphorylation through activation of EGFR and leads to an inhibition of Bax translocation in mitochondria, an important step for apoptosis [51].

PGE2 modulates also tumor growth of lung cancer. This effect has been described by Yamaki et al. [52] showing that PGE2 activates Src kinase in A549 cells, leading to an induction of cell growth. These cells express EP3 that activates Src (sarcoma) kinase. This study has demonstrated that the activation of Src leads to an activating phosphorylation of STAT3, a transcription factor known to regulate cyclin D1 transcription, an important positive regulator of cell proliferation. Apoptosis can be inhibited because STAT3 regulates the transcription of Bcl-XL, a well-known anti-apoptotic protein [53]. Moreover, Src phosphorylates p27, a protein known to inhibit cell cycle progression especially at the G1/S transition [54]. However, it has been recently shown that this protein plays a dual role as the unphosphorylated form of p27 inhibits the cell cycle, and thus cell proliferation. If phosphorylation occurs on T157 and T198 by PI3K (phosphoinositide 3-kinase), it triggers cell cycle transition by stabilizing the cyclin D1/cdk4 complex [55]. Thus phosphorylation of S10 appears to be important for other phosphorylation steps and it has been hypothesized that Src kinase can play this role [55]. Moreover, it is known that phosphorylation of p27 is responsible also for its degradation by the proteasome [56]. All together these data suggest that PGE2 increases cell proliferation via p27 phosphorylation through EP4 receptors.

Nonsmall lung cancer is characterized by a Ras mutation correlated with a poor prognosis [57]. Activation of Ras leads to an upregulation of COX-2 resulting in increased PGE2 production [58]. PGE2 increases cell proliferation of A549 cells (adenocarcinoma) and this effect is associated with an activation of Ras pathway via EP4 receptor. In this case, PGE2 mediates its effect by the release of amphiregulin, the most abundant ligand in A549 cells [59]. EGFR activation leads to activation of MAPK pathway that regulates cell proliferation by transactivating several oncogenes such as c-myc [60].

PGE2 is also important for tumor invasion. A study by Ma et al. [61] described that PGE2 can increase the number of metastasis. This effect has been demonstrated in a model in which murine mammary tumor cells 66.1 were injected in syngenic immune competent BALB/CByJ mice. All these cell lines express EP1, 2, 3, and 4. The use of EP4 antagonists (AH23848 and AH6809) decreased surface tumor colonies and reduced tumor invasion. Another study has revealed that PGE2 increases the level of VEGF in granuloma [62]. VEGF is an important factor of angiogenesis, and thus of tumor progression by enhancing the vascularization of the tumors [63].

Alltogether these data together suggest that PGE2 and, thus, COX-2 play an important role in tumor progression by enhancing cell proliferation, cell survival, and tumor invasion. The diversity of PGE2 receptors and their different signaling pathways suggest that the protumorigenic effect of PGE2 depends on the cell type and the type of receptor expressed. Until now, many signaling pathways associated with tumor progression are linked to PGE2 and this could explain why the use of COX-2 inhibitors is a good strategy in cancer therapy. However, the signaling pathways of EP receptors are not completely characterized and their precise roles in the different cancers remain to be elucidated before a clinical application.

COXs may be targets of several compounds that may inhibit their functions. Combination of such preferential or selective COX-2 inhibitors with anti-cancer agents already used in clinics were tested with the goal to improve the efficiency of anti-cancer protocols. 

**Figure 2:**
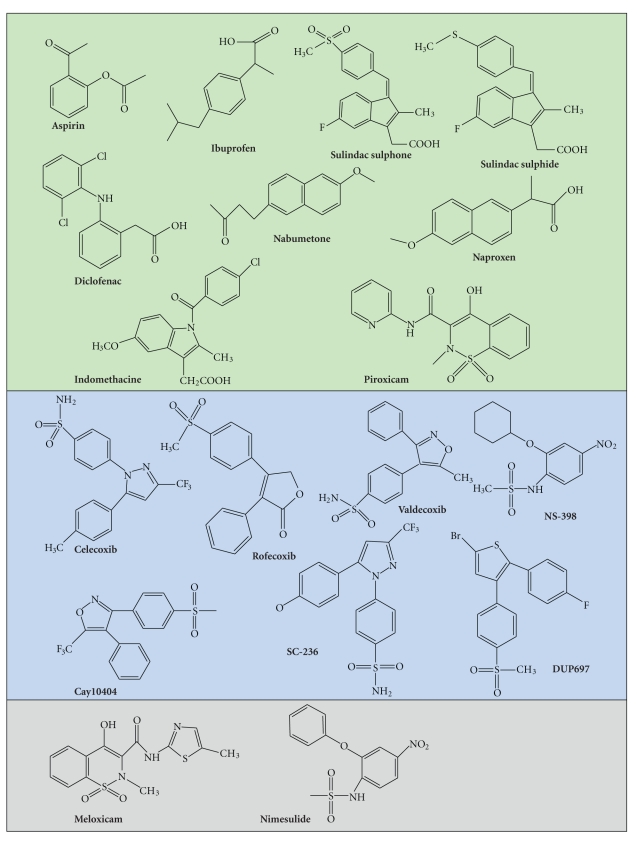
*COX-2 inhibitor classification.* COX-2 is the target of many compounds. COX-2 inhibitors described in this review are classified according to their ability to inhibit COX-2: nonselective (green), selective (pale blue), and preferential (grey).

COX-2 is the preferential target of several NSAIDs (Figure 2) [64, 65]. Historically, NSAIDs used for clinical and anti-inflammatory purposes were represented by the nonselective COX-2 inhibitors, to which belong aspirin, sulindac acid and, more recently, agents such as nimesulide, ibuprofen and naproxen. As their definition well reflects, this first generation of NSAIDs may affect both main COXs isoforms, even if preferentially COX-2 (see above). Their mechanisms of action are not all completely elucidated, complicated by the fact that different agents seem to act in different ways. For example, different NSAIDs bind the active site of COX-2. Commonly, binding occurs by a reversible competitive inhibition (i.e., ibuprofen, naproxen, and indomethacin). In contrast, aspirin is able to acetylate the active site of COX at a serine residue, leading to an irreversible inhibition (see Figure 2, summarizing the classification of COX-2 inhibitors mentioned in this review). Considerable side effects generated by the interference with homeostatic functions modulated by COX-1 include increased incidence of gastrointestinal hemorrhage and ulceration upon chronic or long-time intake [66]. A novel generation of COX-2-selective inhibitors NSAIDs termed “Coxibs” was then developed. These compounds promised to be much less gastrotoxic. They act as competitive inhibitors of the active site of COX-2 and present indeed a higher specificity. However, concerns related to a long-time/chronic intake of these drugs raised quite soon, following some clinical reports, suggest a correlation between an increased risk of myocardial infarction and their consumption [67]. This has lead to the voluntary withdrawal of some of these agents, that is, rofecoxib and valdecoxib [68], and drastic regulatory advices regarding the use of the other ones, thus opening a discussion on the real benefits versus side effects of their use in clinics. Consequently, studies focused on the use of traditional versus COX-2-selective NSAIDs, frequently associated to the elaboration of economical models, have been performed in these latest years, with the aim to evaluate the real risks together with the costeffectiveness and, possibly, identify classes of users/patients where regular NSAIDs intake may be beneficial. Although, further analyses need to be performed, a number of reports suggest that Coxibs may really increase cardiovascular risks only in patients presenting a positivity to other cardiovascular factor risks, as high blood pressure and altered lipid metabolism [69–73]. These results suggest that their use should be limited to patients with a low risk of cardiovascular complications after analysis of multiple biomarkers [Chaiamnuay et al., 2006, clinical reviews]. Therefore, the future perspective in the pharmacological use of preferential versus selective COX-2 inhibitors is the identification of a panel of interesting biomarkers, helping in defining individual biological risk factors and limiting the use of a specific class of COX-2 inhibitors to the appropriate responders [74, 75]. This approach will have a considerable implication in therapy as well as in chemoprevention of inherited forms of colon cancer.

It is interesting to mention that recent alternative approaches have been considered. Strillacci et al. [76] and Chan et al. suggested RNA interference using adenoviral vehicles. Moreover, other selective COX-2 inhibitors have been developed and experimentally used: SC-558 [35], DUP-697 [77], SC-58125 [78], and NS-398 [8]. Some of them induce an irreversible inhibition. This is the case for NS-398, which acts by inducing a conformational change of COX-2 [25] (Figure 2). Another strategy discussed in literature could be the use of EP receptor antagonists. Indeed, it has been demonstrated that EP antagonists can decrease cell proliferation and cell invasion [47, 61, 79]. This could be a more specific strategy that could limit the other side effects of classic COX-2 inhibitors.

## 4. COX-2 As a Regulator of Cell Proliferation

Cell cycle is regulated by different serine-threonine kinase proteins called cyclin-dependent kinase (Cdk). These proteins regulate the different steps of cell cycle progression by phosphorylating many substrates (i.e., nuclear lamins) [54]. These proteins are regulated by phosphorylation and dephosphorylation. Thus, Cdks can be activated by phosphatases such as CDC25C (*cell division cycle 25 homolog C*) for CDK1 or kinase like CAK (Cdk activating kinase). The activity of cdks is also regulated by cyclins, which form heterodimers with cdks leading to an activation of Cdks by conformational change [54, 80].

Cell cycle is under the control of other factors, implicated in the regulation of cell cycle transition. These regulatory mechanisms form checkpoints where the cell cycle can be stopped after cellular damage in order to allow repair and to maintain cellular integrity or, alternatively, to eliminate mutated and potentially dangerous cells. The INK4 family (p16, p15, p18, and p19) and the Cip/Kip family (p21, p27, and p57) [54, 80, 81] are key regulators of G1/S transition. For example, after DNA damage, p53, a tumor suppressor gene, activates transcription of p21, which inhibits cyclin E phosphorylation leading to hypophosphorylation of retinoblastoma protein (pRb) [81]. INK4 family inhibits Cdk4 and Cdk6, whereas Cip/Kip family inhibits all Cdks. Retinoblastoma protein needs to be phosphorylated in order to release transcription factor E2F activating genes involved in the S phase-like PCNA (proliferating cell nuclear antigen) [82]. p53 is also important for the regulation of the G2/M transition, which requires activation of the cyclin B-cdk2 complex. This complex accumulates during the previous step of the cell cycle but is inactivated by a phosphorylation at tyrosine 15 and threonine 14 by Wee 1 and Myt 1. These phosphate groups are removed by the phosphatase CDC25A when cells enter mitosis. In the case of DNA damages, p53 is activated and increases the level of p21 that is directly inhibiting cdk2. Moreover, 14-3-3 protein, a transcriptional target of p53, leads to a sequestration of cdk2 in the cytoplasm [83]. Other mechanisms involved in the regulation of the G2 checkpoint or the mitotic spindle checkpoint are reviewed by Stewart et al. [54].

Cancer cells are characterized by deregulation of the cell cycle via alteration of cell cycle controllers (cyclins) and cell cycle regulators (p53) [54], resulting in a perturbation of cell cycle checkpoints.

Currently, there is evidence that prostaglandins produced by COX-2 intervene in tumor cell proliferation as NSAIDs and selective COX-2 inhibitors inhibit proliferation of different cancer cell types expressing COX-2 [30]. NS-398, a COX-2 specific inhibitor, was described to reduce cell proliferation of MC-26 cell line, a highly invasive mouse CRC cell model expressing constitutively COX-2 [8]. This effect was associated with a reduction of cyclin D level, a key protein involved in G1-S transition [54], and PCNA, thus increasing the processivity of DNA polymerase [82]. NS-398 and COX-2 specific inhibitor nabumetone reduced cell proliferation of U937 (acute promonocytic leukemia) and ML1 (human myeloblastic leukemia), thus leading to an accumulation in G0/G1 phase [33]. Interestingly, meloxicam was also able to downregulate PCNA and cyclin A in HepG2 cell line (hepatocellular carcinoma cells), leading to an inhibition of the cell proliferation and an accumulation of the cells in G0/G1 phase of cell cycle [84]. Alternatively, the link between COX-2 and CRC has been demonstrated by the fact that prostaglandin E2 (PGE2) derivating from COX-2-mediated arachidonic acid metabolism increased the proliferation of colorectal cancer cells [85]. 

The inhibitory effect of NSAIDs on cell proliferation of CRC has been also observed in ovarian cancer. Indeed, treatment of OVCAR-3 tumors xenotransplanted in nu/nu mice (nude mice) with aspirin and piroxicam (NSAIDs) and the selective COX-2 inhibitor meloxicam led to a reduction of tumor growth [86]. 

It has been estimated that 40% of breast cancers show an overexpression of COX-2, which is associated with a bad prognosis [5]. Indomethacin (NSAIDs), celecoxib, rofecoxib and nimesulide have been shown to able to inhibit cell proliferation of these cells [5]. Moreover, prostaglandins were able to increase cell proliferation of hormonal-dependent breast cancer by increasing transcription of CYP19 aromatase implicated in estrogen biosynthesis [87].

**Figure 3:**
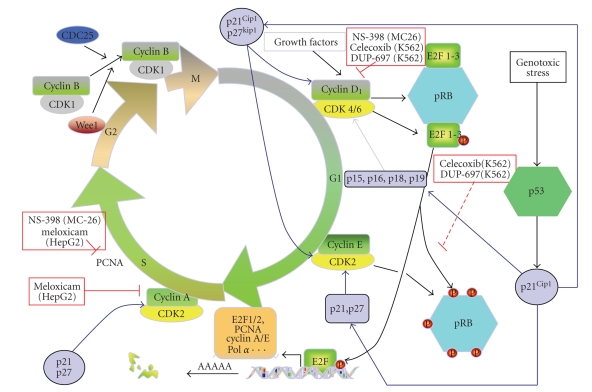
*Effects of COX-2 inhibitors on cell proliferation.* Cell cycle is divided into different steps: G1, S, G2, and M (mitosis). This process is regulated by cyclin proteins, which activate cyclin-dependent kinase (cdk) and phosphatase (i.e., CDC25) or kinase like cyclin-dependent kinase inhibitors such as p16, p15, p18, p19, p21, and p27 [54]. Selective COX-2 inhibitors are able to modulate some cell cycle checkpoints. In this picture, some examples of this link have been shown for different cell types: MC26, colorectal cancer; HepG2, hepatocellular carcinoma; K562, chronic myeloid leukemia. Cdk; cyclin-dependent kinase; pRb, retinoblastoma protein; PCNA, proliferating cell nuclear antigen.

Several studies revealed that inhibition of COX-2 by celecoxib in Burkitt's lymphoma cell lines RAJI, BjAB, (Epstein-Barr virusnegative), and BL41 led to a reduction of cell proliferation [34]. NS-398 and celecoxib were able to reduce proliferation of pancreatic cancer cell line, Panc-1 in a dose-dependent manner [88]. Treatment with celecoxib of these cells implanted into nude mice led to a reduction of the volume of the tumor [88]. Other studies have shown that celecoxib is able to reduce cell proliferation of the chronic myeloid leukemia (CML) cell line K562, which expresses COX-2 at the mRNA and protein level [89]. This effect was accompanied by an accumulation of cells in G0/G1. Moreover, the inhibition of cell proliferation was correlated to a downregulation of cyclin D1, cyclin E, and pRb and the upregulation of p16 and p27 [89]. Similar results were found on this cell type with the other selective COX-2 inhibitor DUP-697 [77]. Different effects are recapitulated in Figure 3.

## 5. Implication of COX-2 in Cell Death

### 5.1. Apoptosis

Apoptosis (type I cell death) is important for the development and maintenance of tissue homeostasis of multicellular organisms [90, 91]. This active form of cell death is characterized by the occurrence of typical cell alterations including plasma membrane blebbing, cell shrinkage, chromatin condensation and nuclear fragmentation, and, finally, formation of apoptotic bodies, which can be phagocyted by macrophages [92]. Deregulation of apoptosis is linked to several pathophysiological disorders, including autoimmune disorders, Alzheimer's disease, and cancer [93]. 

**Figure 4:**
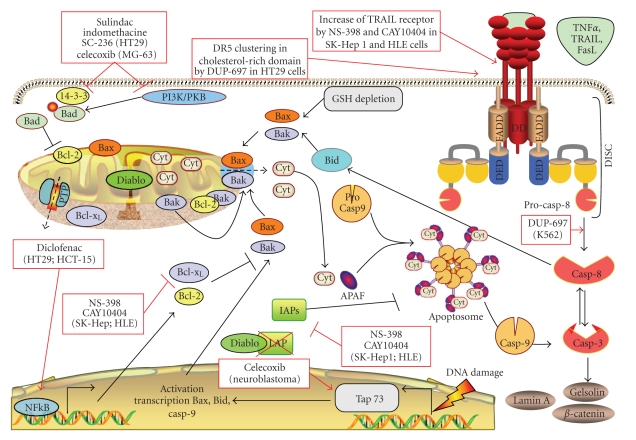
*Effects of COX-2 inhibitors on apoptosis.* Apoptosis can be mainly mediated by two pathways: the mitochondrial, intrinsic, or stress-induced apoptosis, which is activated in response to damaging stresses and the extrinsic pathway, triggered by the binding of ligands to specific death receptors [51]. COX-2 inhibitors are able to modulate stress-induced apoptosis as well as extrinsic apoptosis in several cell types. In this picture, some examples of these interaction discussed in the text are presented for different cell types: LNCaP, prostate cancer; K562, chronic myeloid leukemia; HT29, colorectal cancer; SK-Hep 1 and HLE, human hepatocarcinoma cells; HepG2, hepatocarcinoma; Be17402, hepatocarcinoma; SMMC-7402, hepatocarcinoma; MG-63, osteosarcoma. Abbreviation: AIF: apoptosis-inducing factor; Bcl-2, B cell lymphoma 2; Bid, Bcl-2 interacting domain; Casp, caspase; Cyt, cytochrome C; DD, death domain; DED, death effector domain; DISC, death-inducing silencing complex; PI3K/PKB, phosphatidyl inositol-3 kinase/protein kinase B; FADD, Fas-associated death domain; GSH, glutathione; PTP, transition permeability pore; TNF, tumor necrosis factor; TRAIL, TNF-related-inducing-apoptosis-ligand.

Two major cascades of intracellular events are commonly involved in mediating apoptosis (Figure 4). The intrinsic pathway, also called the mitochondrial or stress-induced apoptotic pathway, is activated in response to damaging stresses, such as DNA damage. Typical hallmarks of this pathway are mitochondrial outer membrane permeabilization (MOMP), accompanied by a collapse of the mitochondrial membrane potential [51]. These events lead to the release of cytochrome *c* into the cytosol, which is an indispensable component of the apoptosome, the death complex formed also by APAF-1, and procaspase-9. Once recruited, this protease is cleaved to its activated form (caspase-9) to further activate the executor caspase-3 and, finally, to finalize the apoptotic program. 

Alternatively, the extrinsic, or physiological, apoptotic pathway (Figure 4) can be triggered upon binding of specific ligands to death receptors characterized by the presence of a death effector domain [94]. Ligands include cytokines, such as TNF*α*, tumor necrosis factor-related apoptosis-inducing ligand-induced apoptosis (TRAIL), or FAS. After binding, death inducing silencing complex (DISC) is formed. The DISC is composed by the adaptors proteins TRADD (TNF receptor-associated death domain) and FADD (Fas-associated death domain) and is able to recruit and activate pro-caspase-8. Finally, caspase-8 activates caspase-3 in order to trigger the final steps of apoptosis (Figure 4).

Cross-talks between the two pathways take place. The extrinsic apoptotic pathway can activate the intrinsic pathway via truncation of the BH3-only protein Bid (t-Bid) by caspase-8. t-Bid interacts with mitochondria, by favoring the activation of the proapoptotic Bcl-2 family members Bak and Bax, thus leading to MOMP and caspase-9 activation [51, 95] (Figure 4). The intrinsic apoptotic pathway may, in turn, activate caspase-8, downstream to caspase-3 [96] (Figure 4). Cross-talks represent an important strategy of amplification loops carried out by dying cells to ensure/potentiate cell death. 

#### 5.1.1. Involvement of COX-2 in Intrinsic Apoptosis

When cells are damaged by a variety of chemicals or physical stress (i.e., reactive oxygen species, UV, and ionizing radiation), they undergo apoptosis by triggering the intrinsic apoptotic pathway (Figure 4). This pathway may be associated with a redox disequilibrium, mediated by depletion of glutathione (GSH) [94, 97, 98], required for the activation and translocation to mitochondria of the Bcl-2 pro-apoptotic member Bax [98], which, in turn, forms complexes (oligomers) mediating MOMP and cytochrome *c* release. As Bax, Bak may play the same role [99]. In contrast to Bax, Bak is already present at the surface of mitochondria, normally sequestered in its active monomeric form by the Bcl-2 anti-apoptotic members Bcl-xL and Mcl-1 (see Burlacu for a general overview of the Bcl-2 family members modulation involved in Bax/Bak activation [51]).

Apoptosis is regulated in order to maintain tissue homeostasis. This regulation implicates protein-protein interactions, with some of them counteracting apoptosis. In this view, the interaction between Bcl-2 family pro- and antiapoptotic members represents a crucial and delicate step. Bcl-2 is the best described member of this family preventing Bax activation [51]. Bax can form also a complex with the anti-apoptotic protein Bcl-xL [53] and Mcl-1 [14]. Similarly, Bak activity is monitored by the anti-apoptotic members Bcl-xL and Mcl-1 [51]. The interaction between Bax/Bak and the Bcl-2 family anti-apoptotic members is carefully regulated by the BH-3-only proteins. Another carefully regulated downstream checkpoint of the apoptotic pathway is the activation of caspases. Inhibitor of apoptosis (IAP) family, by directly interacting with caspases (i.e., XIAP, survivin [100]) controls and prevents their activity once cleaved. IAPs monitoring function can be, in turn, counteracted by the pro-apoptotic SMAC/DIABLO, a mitochondrial heterodimer, which is released from mitochondria when MOMP is affected [101]. This interaction favors the induction of apoptosis. 

Imbalance between cell proliferation and apoptosis observed in cancer can be tightly related to an altered function of pro-apoptotic proteins as well as to an up-regulation of anti-apoptotic proteins (i.e., Bcl-2 or IAPs) or a downregulation of tumor suppressor genes (i.e., p53). In addition, the activation of prosurvival pathways (i.e., PI3K/Akt) may be implicated upstream. Inflammation can contribute to this imbalance *via* cytokines secreted in the tumor microenvironment able to activate survival pathways. For example, TNF*α* can induce NF*κ*B, leading to an inhibition of apoptosis [38]. COX-2 seems also to play a role in this process because it is known that COX-2 inhibition is correlated to an increase of apoptosis in several cancer models. NS-398 downregulated Bcl-2 expression in an androgen-sensitive human prostate cancer cell line LNCaP that exhibited a high constitutive level of COX-2 [102]. Similar results have been observed in human colorectal cancer cells (HCA-7 cell line which expresses COX-2) where PGE2 was able to inhibit apoptosis induced by SC58125, a selective COX-2 inhibitor, and increase Bcl-2 expression [46]. Different mechanisms are supposed to explain how COX-2 inhibitors may trigger apoptosis. In a number of studies, COX-2 inhibition was linked to a concomitant increase of intracellular arachidonic acid. In HT-29 human colon adenocarcinoma cell this accumulation led to the induction of apoptosis [103]. The arachidonic acid-induced apoptosis was inhibited by Bcl-2 transfection, indicating a role of arachidonic acid in affecting Bcl-2 intracellular levels [103]. Accumulation of arachidonic acid can affect apoptosis by mediating an increase of pro-apoptotic intra-cellular ceramides caused by activation of sphingomyelinase [104, 105]. Sulindac sulphide, a metabolite of sulindac, also activates sphingomyelinase and enhances the ceramide level in the two human colorectal carcinoma cell lines HCT116 and SW480 [106]. 

COX-2 reduces pro-apoptotic nitric oxide (NO) levels in cancer cells downstream of prostaglandin production [30] (see Brüne et al. [107] for an overview on nitric oxide role in apoptosis). Chang et al. reported that PGE2 prevented apoptosis induced by NGF (nerve growth factor) withdrawal by increasing the level of dynein light chain, an inhibitor of neuronal NO synthase in pheochromocytoma of the rat adrenal medulla PC12 cells, thus leading to decreased intracellular NO levels [108].

More recently, connections between COX-2 inhibitors and p53 family members have been described. For example, celecoxib was shown to able to modulate different isoforms of p73, a p53 family member in neuroblastoma cell lines [109]. p73 encodes many isoforms with different roles. Tap73 is pro-apoptotic and contains a transactivation domain. This isoform is considered as a tumor suppressor gene because it seems to be involved in cell cycle regulation as well as in apoptosis induction [109, 110]. In contrast, DeltaNp73 is anti-apoptotic and lacks the transactivation domain. DeltaNp73 is overexpressed in neuroblastoma, leading to chemotherapy resistance [109]. It has been shown that celecoxib was able to upregulate Tap73 and downregulate DeltaNp73. These data suggest the use of COX-2 inhibitors as p73 modulators in order to improve efficiency of chemotherapy [110]. 

The apoptotic effect of COX-2 inhibitors has been also observed for other tumor cell types, such as in the chronic myeloid leukemia model K562 where DUP-697 induced apoptosis by cell cycle arrest and caspase-8 activation [77].

COX-2 inhibitors can also activate prosurvival pathways. The PI3K/Akt pathway is a survival pathway, frequently activated in cancer cells [49]. PI3K produces PIP3 (phosphatidylinositol 3,4,5 triphosphate) that activates PDK1 (pyruvate deshydrogenase kinase). This protein phosphorylates and activates PKB (protein kinase B), which, in turn, is responsible for the phosphorylation of several targets playing a modulator function in apoptosis. An anti-cancer effect of celecoxib due to the inhibition of Akt signaling [111] was observed in a gastric cancer model. Celecoxib triggered also apoptosis in osteosarcoma cells (MG-63) through down-regulation of Bcl-2, survivin and PI3K (phosphoinositide 3-kinase) pathway [112]. Similarly, Hsu et al. [113] found that inhibition of Akt phosphorylation by celecoxib in prostate cancer models (LNCaP and PC3 cell lines which express constitutively COX-2) led to apoptosis, but in this case without affecting Bcl-2 level. 

The PI3K pathway is negatively regulated by PTEN (phosphatase and TENsin homolog), which converts PIP3 in PIP2, preventing PKB activation and Bad phosphorylation/sequestration. Thus PTEN is considered as a tumor suppressor gene. It has been shown that NS-398 was able to increase the level of PTEN in human gastric carcinoma cell line MKN45 [114]. 

One of the PKB targets is Bad, a BH3-only member [51]. The nonphosphorylated form of Bad plays a pro-apoptotic role, by binding Bcl-xL or Bcl-2 and, thus, preventing their interactions with Bak and Bax. The activation of PI3K/Akt pathway may lead to the phosphorylation of Bad, which is consequently sequestrated in the cytoplasm by 14-3-3 protein and, in this way, inhibited in its pro-apoptotic function [51]. It has been reported that sulindac sulphone, indomethacine, and SC-236 were able to induce apoptosis *via* Bad activation, by inhibiting 14-3-3 expression in a dose- and time-dependent manner in HT-29 cells [115]. This effect was tightly related to PPAR*δ*. It is known, indeed, that 14-3-3 protein contains PPRE recognized and bound by PPAR*δ*  [115]. COX-2 can mediate the synthesis of prostaglandin I2, which can bind and activate PPAR*δ*  [41]. Thus, it has been suggested that the inhibition of COX-2, leading to a decrease of PGI2, impaired PPAR*δ* activation, which, in turn, was responsible for a downregulation of 14-3-3 protein, thus allowing Bad to play its pro-apoptotic functions [115].

NF*κ*B is a most important transcription factor involved in survival by enhancing transcription of anti-apoptotic proteins such as Bcl-2 [14, 15]. Sulindac inhibits NF*κ*B in two colon cancer cell lines (human colon adenocarcinoma HCT-15 and HT29 cell lines) [116]. Similar results were obtained with diclofenac, which was able to inhibit nuclear accumulation of NF*κ*B [117]. In the same study, PGE2 was demonstrated to increase the transcriptional activity of *Ν*NF*κ*B p65/p50 dimer in CACO-2 cells (human epithelial colorectal adenocarcinoma cells), transfected with a luciferase construct containing NF*κ*B response elements [117].

It is known that activation of prostaglandin receptors induces an increased cAMP level which in turn can activate protein kinase A (PKA) [58]. Studies have suggested that PKA, like PKB, phosphorylates Bad [118], leading to its sequestration and inhibition of apoptosis. Some of the pro- or anti-apoptotic mediators affected by COX-2 inhibitors are schematized in Figure 4.

#### 5.1.2. Implication of COX-2 in Extrinsic Apoptotic Cell Signaling Mechanisms

Studies reported that COX-2 inhibitors are also associated with a sensitization of tumor cells to extrinsic apoptosis. Thus, DUP-697 sensitized HT29 colon cancer cell line to TRAIL-induced apoptosis. This effect was due to an accumulation of arachidonic acid inside the cells, which activates sphingomyelinase, triggering a clustering of death receptor (DR) 5 receptors in ceramide and cholesterol-rich domains [119]. Alternatively, the expression of COX-2 has been frequently associated with a modulation of the expression of death receptors, thus leading to an upstream control of the extrinsic apoptotic pathway. Tang et al. [120] showed that COX-2 overexpression in human colon cancers cells led to an inhibition of DR5 expression and a resistance to TRAIL-induced apoptosis. Accordingly, COX-2 specific inhibitors, NS-398 and CAY10404, are sensitizing human hepatocarcinoma cells (SK-Hep1 and HLE) to TRAIL-induced apoptosis. This effect was due to an upregulation of TRAIL receptors (TRAIL R2/DR5 and TRAIL-R1/DR4), together with an ability of the compounds to induce a down-regulation of the anti-apoptotic proteins survivin (IAP) and Bcl-xL [121]. In hepatocellular carcinoma models (HepG2, Bel7402, and SMMC-7402), Li et al. [84] showed that COX-2 inhibition with meloxicam led to an upregulation of Fas-mediated apoptosis. In vivo studies performed on transgenic mice constitutively expressing human COX-2 confirmed an increased resistance to Fas-induced apoptosis in liver, as shown by the preservation of liver architecture in COX-2-expressing mice compared to wild type [122]. Similarly, another study performed on human extrahepatic bile duct carcinoma cell line showed that COX-2 induction led to the inhibition of Fas-induced apoptosis, whereas the inhibition of COX-2 with NS-398 in cytokine-treated cells exacerbated apoptosis induced by CH-11, an agonist of Fas receptor [123].

AKT pro-survival pathway may play a role also in the modulation of extrinsic apoptosis. The human gastric carcinoma cell line MKN45, which expresses COX-2, was sensitized to Fas-induced apoptosis by NS-398. The COX-2 inhibitor, indeed, was able to increase the level of PTEN, leading to a decrease of Akt phosphorylation and activation of Bad [114]. Some effects of COX-2 inhibitors on extrinsic apoptosis are summarized in Figure 4.

Altogether, these results encourage the perspective that COX-2 inhibitors could be used in future as a therapeutic strategy to sensitize tumor cells to apoptosis induced by physiological stimuli.

### 5.2. Involvement of COX-2 in Other Types of Cell Death

#### 5.2.1. Anoikis

Anoikis is a form of apoptosis mediated by the loss of cell anchorage. This pathway plays a fundamental role during development and maintenance of tissue homeostasis by killing damaged cells or detached cells in order to maintain tissue architecture. For example, the inner endodermal cells undergo anoikis after the loss of anchorage to the matrix during development [124]. It is known also that intestinal epithelial cells loose anchorage when located at the luminal surface, leading to anoikis [42]. As a form of apoptosis, anoikis is dependent on caspase activation and cytochrome *c* release by mitochondria and is regulated by Bcl-2 family members [42]. 

It has been shown that anoikis is prevented in cancer cells, thus favoring tumor progression with the formation of metastasis [42]. Accordingly, modulation of anoikis is considered a promising target for anti-cancer strategies.

Cell anchorage is due to cell-cell and cell-matrix interactions. Cell-cell interactions are mainly mediated by integrins which are transmembrane receptors located at the cell surface and composed of alpha and beta chains [125]. Many intracellular signals can act downstream to integrins, which, correctly switched on, can ensure cell survival. Some of them are mediated by kinases such as Focal-adhesion-kinase (Fak) or integrin-linked kinase (ILK) [42]. Fak is phosphorylated upon integrin adhesion, leading to activation of other signaling pathways like PI3K, MAPK. ILK is a serine/threonine kinase that directly phosphorylates PKB. 

Together with cell-cell and cell-matrix interactions, paracrine factors could be important for the regulation of anoikis. It has been shown that E-cadherin (epithelial cadherin) can activate COX-2 [23]. It is possible that prostaglandins produced by COX-2, which act in an autocrine and a paracrine manner, favor cell survival. A study from Joseph et al. [126] showed that PGE2 inhibited anoikis in IEC-18 cells (rat intestine ileum cells). This effect was suggested to be due to cAMP signaling because prostaglandin E2 receptors are coupled to adenylate cyclase, which converts AMP to cAMP [126].

Other studies demonstrated that COX-2 inhibits anoikis via activation of PI3K/Akt pathway, as the case of a human bladder cancer cell line expressing COX-2 [40]. A link between COX-2 and anoikis has been described, furthermore, in uterine endometrial carcinoma [127]. COX-2 is over-expressed in this type of cancer and this is associated with tumor aggressiveness. In addition, a recent report based on HEC-1B and RL95-2 (two human endometrial cancer cell lines) showed that the treatment of these cells with hepatocyte growth factor (HGF) led to an up-regulation of COX-2. Hepatocyte growth factor interacts with its tyrosine kinase receptor c-Met. This interaction is responsible for tumor progression. Overexpression of HGF/c-Met has been described in different tumors such as breast cancer [128] as well as head and neck cancer [129], also in endometrial carcinoma [130]. It has been demonstrated that HGF inhibited anoikis and treatment of HEC-1B and RL95-2 cells with the COX-2 selective inhibitor meloxicam prevented HGF-mediated anoikis resistance [127]. Similar results were obtained in head and neck squamous cell carcinoma [131]. 

Altogether these data suggest that COX-2 may be implicated in the inhibition of anoikis and that COX-2 inhibitors may play a role in inhibiting tumor progression (metastasis), by sensitizing tumor cell to anoikis.

#### 5.2.2. Autophagy

Autophagy is a process triggering cells to degrade intracellular constituents, ranging from proteins up to entire organelles. It represents an important process ensuring the turnover of long-lived cellular components, which can be activated also by stress conditions like nutrient starvation in order to avoid cell death. The process starts with the formation of doubled membrane-bound vacuoles corresponding to autophagosomes that entrap parts of the cytoplasm or organelles (i.e., mitochondria). Then, these structures are fused with lysosomes (autolysosomes), thus leading to the degradation of the intracellular parts previously enclosed. Together with apoptosis, when exacerbated, autophagy contributes to the modulation of homeostasis, by eliminating damaged and potentially dangerous cells (type II cell death) [132]. However, the relationship between apoptosis and autophagy is currently still poorly understood [132] because in some cases autophagy permits an adaptation of the cells to stress (i.e., nutrient starvation), thus counteracting apoptosis, whereas, in other cases, autophagy is a process triggering downstream apoptosis [132]. Indeed, similar stimuli can induce both apoptosis or autophagy [132]. 

This process is implicated in pathologies such as Alzheimer's disease and cancer, suggesting a promising field in therapy. By considering that COX-2 is supposed to play a role in apoptosis and a link between apoptosis and autophagy exists, it is conceivable that COX-2 plays a role also in this process. Currently, not many studies aimed at investigating a possible link between COX-2 and autophagy have been published. Nevertheless, one study revealed that sulindac sulphide (NSAIDs) induced apoptosis of the colon cancer HT29 cell line. This effect was increased by treatment of the cells with 3 methyl-adenine, a well-known inhibitor of autophagy [133]. Moreover, the extent of apoptosis in Q204L cells (a clone of HT-29 cells in which 3 methyl-adenine-sensitive autophagic sequestration is impaired) was less than in HT29. These data suggest that autophagy can delay sulindac sulphide-induced apoptosis [133].

## 6. COX-2 Inhibitors in Cancer Therapy

Despite the latest progress in cancer research and the different strategies to kill cancer cells, several tumors are resistant to conventional therapeutics treatment (i.e., radiotherapy, chemotherapy, and photodynamic therapy).

COX-2 inhibitors play an important role in cancer prevention. Indeed, the chronic intake of NSAIDs is able to consistently reduce the appearance and incidence of many types of cancer as described in Familial Adenomatous Polyposis (for celecoxib) [134, 135] and also in breast cancer [136]. This property of COX-2 inhibitors could be useful for patients with a high risk to develop cancer such as people with Li-Fraumeni syndrome, for example [137]. The fact that many reports in literature suggest that COX-2 inhibitors are responsible for an inhibition of cell proliferation and apoptosis induction in a number of different cancer cell models prompts to consider a possible use of COX-2 inhibitors in future therapeutical protocols, administered alone as well as in combination with anti-cancer clinical protocols in order to improve tumor cell death. 

### 6.1. COX-2 Inhibitors in Combination with Radiotherapy

Radiation therapy is a common treatment used for the treatment of solid tumors, such as breast, prostate, colorectal, and lung cancers. It is known that the anti-cancer properties of ionizing radiation are due to pleiotropic mechanisms. Radiation leads to the formation of DNA doubled-strand breaks in proliferating cells, which triggers the activation of DNA damage pathways (i.e., p53), followed by the induction of apoptosis [36]. The importance of Bcl-2 family members during apoptosis [51] suggests that prosurvival proteins (i.e., Bcl-2, Bcl-xL) play an important role in radioprotection of tumor cells. The NF*κ*B pathway seems to be implicated, being required in regulating expression of the anti-apoptotic Bcl-2 family members like Bcl-xL [36]. Moreover, it is well established that NF*κ*B regulates the level of COX-2, suggesting that COX-2 may play a role in radiotherapy resistance [21]. Similarly, nimesulide could increase radiation efficiency in nonsmall cell lung cancer in vivo (nude mice) and in vitro (A549 cell line) as shown by Grimes et al. [138]. This effect was due to a down-regulation of MnSOD (superoxide dismutase containing manganese (Mn) and localized in mitochondria), a primary antioxidant protein and survivin, an anti-apoptotic protein (IAPS family member). These two proteins are regulated by NF*κ*B. It is well known that during radiation therapy NF*κ*B can be upregulated due to reactive oxygen species release and inflammation (i.e., PGE2). This report suggests that nimesulide may act on NF*κ*B to inhibit MnSOD and survivin. 

Melanoma is known to be very resistant to conventional radiotherapy and chemotherapy. Irradiation of two melanoma cell lines WM35 and LU1205 in the presence of NS-398, a selective COX-2 inhibitor, strongly exacerbated the G2/M arrest as well as the induction in apoptosis. Accordingly, the down-regulation of COX-2 by RNA interference in these cell lines was followed by an upregulation of p53 and G2/M arrest [36], thus confirming that the effect of NS-398 is due to its role on COX-2 inhibition. 

Other studies have shown that the radiosensitivity of PC3 (human prostate carcinoma cells) and Hela (human cervical carcinoma cells) was enhanced after silencing of COX-2 by siRNA. NS-398 was able to increase radiosensitivity of PC3 cells expressing COX-2, but not in PC3 silenced for COX-2. In contrast, NS-398 enhanced radiosensitivity of Hela cells, irrespective to the level of COX-2 [37]. 

However, combination of COX-2 inhibitors with radiation therapy can also lead to a reduction of efficiency of the radiotherapy. In one report, it has been shown that the selective COX-2 inhibitor nimesulide decreased radiation efficiency of two head-and-neck cancer cells lines (SCC9 and SCC25) which are COX-2 positive [139]. This suggests that the sensitization of tumor cells to radiation might be strongly dependent on tumor cell type.

### 6.2. COX-2 Inhibitors in Combination with Chemotherapy

Many types of cancer are treated with chemotherapeutic agents leading to inhibition of cell proliferation or induction of apoptosis [140].

One of the major causes of chemotherapy failure is the survival and/or development of multidrug resistant cancer cells. This resistance is mediated by many mechanisms including over-expression of proteins involved in inhibition of apoptosis (i.e, Bcl-2), leading to insensitivity of tumor cells to apoptotic stimuli; an up-regulation of DNA repair; alteration of the target; up-regulation of detoxification enzymes (i.e., Glutathione S-transferases); and extrusion of chemotherapeutic drugs by overexpression of ATP-binding cassette family proteins, such as MRP (multidrug resistant-associated protein) BCRP (breast cancer resistance protein or mitoxantrone resistance protein) because these proteins regulate absorption, distribution, and excretion of various pharmacologic compounds [141]. Consequently, the chemotherapeutic agents are immediately extruded from the cells. P-gp (P-glycoprotein) is one of the best-understood mechanisms leading to multidrug resistance (MDR). Tremendous efforts have been made to find solutions to overcome MDR. Recently, COX-2 inhibitors showed an ability to sensitize tumor cells to chemotherapeutic agents in several models and also in clinical assays. Colorectal cancers are particularly affected by chemoresistance. One study revealed that the COXs inhibitors naproxen and indomethacin heptyl ester were able to downregulate P-glycoprotein in human colorectal CACO-2 cell line. [39]. Indomethacin inhibited the activity of the protein and affected COX-2 mRNA and protein level [39]. Another study showed that meloxicam was able to downregulate MDR1 in HL60 (a human promyelocytic leukemia) cell line as well as in acute myeloid leukemic blasts [142]. The regulation of MDR1 by COX-2 has been also suggested in another study [143] in which it was reported that transfection of COX-2 cDNA with adenovirus in renal rat mesangial cells led to an upregulation of MDR1 gene. The combination of COX-2 inhibitors with chemotherapy was also assayed in a study in which the sensitivity of a human gastric cancer cell line MKN45 to cisplatin (alkylating agent) resulted increased by COX-2 downregulation with siRNA [35], suggesting a possible therapeutic application of this combination. Similarly, the sensitivity to cisplatin was increased by celecoxib in a human osteosarcoma cell line (MG-63) and this effect was linked to a down-regulation of anti-apoptotic proteins survivin, Bcl-2, and an inhibition of the survival pathway PI3K/Akt [112]. It was also reported that B-CLL (B chronic lymphoid leukemia) overexpressed COX-2 and the combination of NS398 with chlorambucil, an alkylating agent, increased the level of apoptosis in B-CLL blasts coming from patients [32]. Moreover, several lymphoma cell lines overexpressed COX-2, such as RAJI, BJAB, BL41 and treatment of these cells with celecoxib led to a decrease of cell proliferation in a dose-dependent manner [34]. 

NS-398 was able to increase the cytotoxicity of gemcitabine, an analog of the antimetabolite nucleoside deoxycytidine, used for treatment of nonsmall cell lung carcinoma, in A549ACA cell line (lung adenocarcinoma cell line) by enhancing apoptosis [144]. The combination of NS-398 and gemcitabine is also associated with an inhibition of cell proliferation with an accumulation of the cells in G0/G1 phase of cell cycle and an increase of p21 [144].

All of these data suggest that COX-2 is implicated in anti-apoptotic and MDR pathways and that selective COX-2 inhibitors could be used to improve chemotherapy efficiency.

### 6.3. COX-2 Inhibitors in Combination with Photodynamic Therapy

An alternative therapeutic approach to treat cancers is photodynamic therapy. This procedure is particularly used for such solid tumors including skin, bladder, and head and neck cancers in addition to other diseases like age-related macular degeneration and psoriasis [145]. The treatment consists in the administration of a photosensitizer, a molecule that selectively accumulates in tumors and is activated by light (600–850 nm). The photosensitizers may accumulate in different compartments of tumor cells like mitochondria (i.e., porphycene monomer), nucleus, lysosomes (i.e., lysyl chlorin p6), and plasma membrane (i.e., monocationic porphyrin like Photofrin). Then, the photosensitizer is excited with a laser from a single state to a triplet state. The triplet-state photosensitizer is implicated in two oxygen-dependent reactions. In the first one, the triplet can react with cell membrane or molecules, leading to radical formation, which in combination with oxygen produce oxygenated products, cytotoxic for the cells [146, 147]. In the second reaction, the triplet-state photosensitizer can transfer its energy directly to oxygen in order to produce singlet oxygen (^1^02), which is known to be a very highly reactive oxygen species and is implicated in cell damage. Therefore, this therapy leads to tumor destruction due to cell death occurring *via* apoptosis and necrosis. Vasculature damages and activation of immune response are two important effects implicated in tumor ablation.

Some parameters affect PDT efficiency, such as the distribution of the photosensitizer, photobleaching, hypoxia/anoxia, and the vascularization of the tumor [146]. The main reason of failure of this therapeutic approach is linked to an up-regulation of angiogenic and inflammatory factors in the tumor microenvironment that strongly reduces the PTD efficiency with a consequent tumor relapse. The link between inflammation and survival pathway activation, cell proliferation, and angiogenesis is well known and contributes to tumor progression [3, 6]. It has been shown that PDT leads to an increase of TNF*α*, IL1*β*, PGE2, VEGF (vascular endothelial growth factor), and MMP9 (matrix metalloproteinase 9) [147]. These molecules can counteract tumor responses to PTD by promoting cell proliferation and cell survival [38]. Interestingly, it has been demonstrated that COX-2 is upregulated during PDT treatment in different cancer models. As for radiotherapy and chemotherapy, this suggests COX-2 as a possible target to increase PDT efficiency. 

Indeed, celecoxib has been proved to affect the Photofrin-induced PDT in in vitro and in vivo studies performed on a mouse mammary carcinoma BA cell line [148]. In vitro, celecoxib and NS-398 increase PDT-induced apoptosis. These results were correlated with caspase-3 and PARP cleavage and Bcl-2 degradation. In vivo, the photosensitization by COX-2 inhibitors was not due to apoptosis exacerbation. Interestingly, celecoxib and NS-398 decrease PDT-induced apoptosis but were also able to decrease the level of angiogenic factors such as TNF*α*, IL1*β*, PGE2, VEGF, and MMP9 [148].

Upon chlorin-induced PDT, COX-2 was found up-regulated 25-fold in mouse mammary carcinoma RIF cells [149]. This up-regulation was associated with an increase of PGE2 level in the tumor microenvironment. When RIF cells were transplanted in CH3/HeJ mice, for in vivo studies, PDT similarly induced an increase of COX-2 and PGE2. These effects were prevented by NS-398. Here, it was demonstrated that PDT induced vascular endothelial growth factor expression (VEGF) and this increase was attenuated by treating mice with NS-398, meaning that COX-2 might play a role also in angiogenesis. In consequence of these effects, the combination of COX-2 inhibitors with PDT resulted in an increased efficiency of tumor treatment. 

Possible correlation between COX-2 level and resistance to PDT has been also investigated in Hela (human cervix carcinoma cells) and T24 (human transitional cell carcinoma of the urinary bladder) cells [150]. It has been reported that in PDT induced by hypericin, a natural photosensitizer which accumulates in endoplasmic reticulum and Golgi apparatus, an increase of PGE2 levels occurred. Hypericin induces apoptosis by triggering the release of cytochrome *c* after light excitation through a process requiring the activation of p38 MAPK, which it is known to induce an up-regulation of COX-2 [23, 151]. The increase in PGE2 levels was prevented by the use of a p38 MAPK inhibitor (PD169316). Moreover, the impairment of p38 MAPK was associated with an increase in the susceptibility of tumor cells to PDT. However, COX-2 inhibitors did not lead to the same effect, meaning that COX-2 was not involved in PDT resistance in this model.

In contrast to the study of Ferrario et al. [148, 149], a report from Makowski et al. [152] has revealed that rofecoxib, NS-398, and nimesulide were unable to potentiate PDT in C-26 cells (poorly differentiated colon adenocarcinoma cell line) in vitro. However, chronic exposition of mice bearing C-26 cells to nimesulide potentiated PDT. These data suggest that COX-2 inhibitors may indirectly potentiate PDT. 

It is known that vasculature damages are important for PDT efficiency and that COX-2 inhibitors act as anti-angiogenic factors [153]. It has been hypothesized that these antiangiogenic effects could be responsible for the anti-tumor effect. 

Currently, the link between COX-2 and PDT efficiency is not well characterized. Some studies have revealed an improvement of efficiency with COX-2 inhibitors whereas other reports have demonstrated no direct effects. In any case, this effect may be cell-type dependent as for chemotherapy or radiotherapy.

## 7. Inhibition of COX-2 Expression by Natural Compounds

Synthetic cyclooxygenase-2 inhibitors hold promise for cancer chemoprevention; however, recent toxicity problems suggest that new strategies are needed. Natural compounds with the potential to inhibit key cell signaling pathways including COX-2 gained much attention over the last regarding years whether they are used alone or in combination with existing chemotherapeutic agents.

Recently, Bhui et al. demonstrated that Bromelain, a pharmacologically active compound present in pineapple (Ananas cosmosus), leads to a marked inhibition of COX-2 expression and inactivation of NF*κ*B. Bromelain treatment induces up-regulation of p53 and Bax and subsequent activation of caspase-3 and caspase-9 with a decrease in Bcl-2 expression [154]. Furthermore bromelain induces apoptosis-related proteins along with inhibition of NF*κ*B -driven COX-2 expression by blocking the MAPK and Akt/protein kinase B signaling in DMBA-TPA-induced mouse skin tumors [155].

Curcumin, a naturally occurring polyphenol from Curcuma longa, was described to act as an antiinflammatory and antiproliferative agent by causing downregulation of COX-2 in cervical cancer. Curcumin-mediated apoptosis in these cells is initiated by up-regulation of pro-apoptotic Bax, AIF, release of cytochrome *c,* and downregulation of anti-apoptotic Bcl-2, Bcl-xL in HeLa and SiHa cell lines. This onset of apoptosis was accompanied by an increase in caspase-3 and -9 activity, suggesting the role of mitochondria in curcumin-mediated apoptotic cell death as described by M. Singh and N. Singh [156]. Marín et al., furthermore, concluded that curcumin inhibits NF*κ*B activity as well as the expression of its downstream target genes, and also selectively induces apoptosis of melanoma cells but not normal melanocytes [157]. In addition, curcumin-induced apoptosis was also associated with the activation of caspase-3 and caspase-9, and the degradation of PARP. Curcumin decreased the expression levels of COX-2 mRNA and protein without causing significant changes in COX-1 levels, which was correlated with the inhibition of prostaglandin E(2) synthesis [158]. In BV-2 microglial cells, curcumin and analogs were shown to inhibit LPS-induced COX-2 expression; analogs identified as more potent than curcumin in the screening assay were also more potent than curcumin in preventing COX-2 expression [159].

Coumarin (1,2-benzopyrone) is a naturally occurring fragrant compound found in numerous plants and spices. Results obtained with human nonsmall cell lung cancer A549 cells suggest that downregulation of Bcl-xL, COX-2, and MAP kinase pathway and up-regulation of p53, Akt, and NF*κ*B pathway are involved in the underlying molecular mechanism of apoptosis induction as suggested by Goel et al. [160]. 

Suh et al. concluded that the plant flavonoid fisetin induces apoptosis and suppresses the growth of colon cancer cells by inhibition of COX-2- and Wnt/EGFR/NF*κ*B-signaling pathways [161].

Sulforaphane (SFN) is a biologically active compound extracted from cruciferous vegetables, and presents potent anti-cancer and anti-inflammatory activities by suppression of NFkB-dependent genes involved in anti-apoptotic signaling (IAP-1, IAP-2, XIAP, Bcl-2, and Bcl-xL), cell proliferation (c-Myc, COX-2, and cyclin D1), and metastasis (VEGF and MMP-9) as published by Moon et al. [162]. 

Nontoxic apigenin can suppress anti-apoptotic pathways involving NF*κ*B activation including cFLIP and COX-2 expression as demonstrated by Xu et al. [88]. According to Nam et al., DA-6034, a synthetic derivative of flavonoid Eupatilin, strongly enhanced apoptosis and inhibited the expression of COX-2 and phospho-IKKalpha in inflammation-related colon cancer models [163].

EGCG from green tea was described to attenuate the AR, to downregulate IGF-1, to modulate COX-2 expression, and to decrease MAPK signaling leading to the reduction in cell proliferation and induction of apoptosis in prostate cancer without toxicity [164]. Interestingly, combination of EGCG and COX-2 inhibitor NS-398 enhanced cell growth inhibition, apoptosis induction, expression of Bax, pro-caspase-6, and pro-caspase-9, and PARP cleavage, inhibition of PPAR gamma, and inhibition of NF*κ*B compared with the additive effects of the two agents alone, suggesting a possible synergism. In vivo, combination treatment with green tea polyphenols and COX-2 inhibitor celecoxib resulted in enhanced tumor growth inhibition, lowering of prostate-specific antigen levels, lowering of IGF-I levels, and circulating levels of serum IGF-1 binding protein-3 compared with results of single-agent treatment. Accordingly, Adhami et al. postulate the efficiency of synergistic and/or additive effects of combinatorial chemopreventive agents and further underscore the need for rational design of human clinical trials involving such natural compounds [165]. 

Pandey et al. published that butein inhibited the expression of the NF*κ*B-regulated gene products involved in anti-apoptosis (IAP2, Bcl-2, and Bcl-xL), proliferation (cyclin D1 and c-Myc), and invasion (COX-2 and MMP-9). Suppression of these gene products correlated with enhancement of the apoptosis induced by TNF and chemotherapeutic agents, and inhibition of cytokine-induced cellular invasion. This group clearly demonstrates that antitumor and anti-inflammatory activities assigned to butein may be mediated in part through the direct inhibition of IKK, leading to the suppression of the NF*κ*B activation pathway [166].

Hostanska et al. used human colon COX-2-positive HT 29 and COX-2-negative HCT 116 or lung COX-2 proficient A 549 and low COX-2 expressing SW2 cells and showed that willow bark extract BNO 1455 and its fractions inhibit the cell growth and promote apoptosis in human colon and lung cancer cell lines irrespective of their COX selectivity [167].

## 8. COX-2 Independent Effects

It is currently well known that several selective COX-2 inhibitors inhibit cell proliferation and induce apoptosis independently of COX-2. Celecoxib is particularly known to have these COX-2-independent effects, which were reviewed by Grosch et al. [68]. Indeed, celecoxib was able to directly bind and inhibit PKB/Akt, which plays an important role in cell proliferation and in apoptosis. Concerning cell cycle, PKB is able to phosphorylate cdk inhibitors, such as p21 and p27, leading to PCNA activation [168, 169]. Furthermore, PKB can also activate several cyclin-cdk complexes and induce E2F factor in some cases [68], stimulating cell proliferation. Besides, PKB inhibits apoptosis, by phosphorylating the pro-apoptotic protein Bad and by inhibiting caspase-9 cleavage [51].

The COX-2-independent effects concern also the extrinsic apoptotic pathway. Indeed, we discussed that selective COX-2 inhibitors, such as NS-398, celecoxib, and meloxicam, are able to modulate the sensitivity of several tumor cells to Fas- and TRAIL-induced apoptosis. It has been discussed that this modulation could be due to COX-2-independent effects. In fact, NS-398 and nimesulide were able to promote TNF and TRAIL-induced apoptosis of D98 and H21 Hela cell lines [170]. In D98, COX-2 is inactive. Moreover, prostaglandin E2 readdition was not able to revert the sensitization effect. In the same report, it has been shown that NS-398 was able to promote apoptosis induced by TNF in MCF-7 cell line (human breast adenocarcinoma cells), which again does not express COX-2 [170].

A report from Ryan et al. [171] demonstrated that SC58125 and CAY10404, two selective COX-2 inhibitors, were able to decrease intracellular content of GSH in malignant human B-cells. This effect was accompanied by an increase of reactive oxygen species production. Indeed, GSH is the most important intracellular nonprotein thiol antioxidant defense against free radicals, meaning that it protects the cells from cellular damages. The GSH depletion was correlated in this study with a reduced survival for these cells.

The fact that many studies imply an association between COX-2 inhibition and apoptosis induction or cell proliferation inhibition, without assessing whether COX-2 activity is effectively decreased, suggests caution in the interpretation of the data. This is confirmed by the observation that different COX-2 inhibitors may trigger apoptosis in the same cancer cell model by modulating different mechanisms. For example, celecoxib [113] induced apoptosis by an inhibition of Akt phosphorylation in prostate cancer cells COX-2-positive LNCaP without affecting Bcl-2 level. In contrast, a study by Liu et al. [102] revealed that NS-398 in the same cell line was able to induce apoptosis but down-regulation of Bcl-2. These results suggest a possible COX-2-independent effect and strongly recommends considering in parallel other experimental strategies to ascertain the effective role of COX-2 in human malignancies [33], such as methodologies based on RNA interference or antisense oligonucleotides. Studies have already suggested these alternative methods. It has been shown that the sensitivity of a human gastric cancer cell line MKN45 to cisplatin (alkylating agent) was increased by COX-2 downregulation with siRNA [35].

## 9. Conclusion

**Figure 5:**
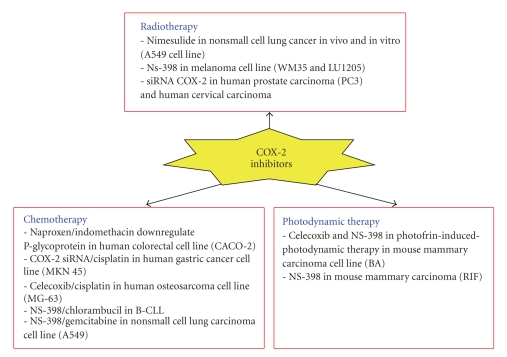
COX-2 inhibition in cancer therapy.

A number of studies suggest that COX-2 inhibition may lead to an inhibition of cell proliferation in different cancer types. The fact that COX-2 inhibition may per se trigger apoptosis of tumor cells and/or sensitize them to cytotoxic treatments is an indication that COX-2 may be a good target in cancer therapy, in order to improve the efficiency of tumor cell death and to reduce tumor progression (see Figure 5 for a synthesis). Accordingly, the combination of selective COX-2 inhibitors with radiotherapy or different chemotherapeutics revealed a sensitization to apoptosis. This effect was also observed with several agents inducing apoptosis in a physiological way, thus suggesting that COX-2 inhibitors used in combination with death receptors agonists might be a novel approach to elicit apoptosis of cancer cells. However, the fact that COX-2 inhibitors can mediate their effects by COX-2-independent mechanisms suggests caution in the interpretation of the data.

Nowadays, selective COX-2 inhibitors have been included in several clinical assays. Some of them effectively increase the efficiency of radiotherapy and chemotherapy [172]. For example, celecoxib is in a clinical phase II assay in combination with Paclitaxel, carboplatin, and radiotherapy for patients with inoperable stage IIIA/B nonsmall cell lung cancer [172]. These clinical assays confirm that COX-2 inhibition may be a promising field in cancer treatment. However, the selective COX-2 inhibitors are responsible for side effects, including an increasing risk of cardiovascular complications [67, 68]. It is hoped that other methods to inhibit COX-2 will be developed. To this purpose, RNA interference using vehicle (i.e., adenovirus) as well as natural compounds were suggested by some studies [35, 76], as alternative and promising strategy.

##  Conflicts of Interest

The authors have no actual or potential conflict of interest.
